# Case Report: Combination of Olaparib With Chemotherapy in a Patient With ATM-Deficient Colorectal Cancer

**DOI:** 10.3389/fonc.2021.788809

**Published:** 2021-12-22

**Authors:** Georgios I. Papageorgiou, Evangelos Fergadis, Nikos Skouteris, Evridiki Christakos, Sergios A. Tsakatikas, Evangelos Lianos, Christos Kosmas

**Affiliations:** Division of Medical Oncology & Hematopoietic Cell Transplant Unit, Department of Medicine, ‘‘Metaxa’’ Cancer Hospital, Piraeus, Greece

**Keywords:** PARP inhibition, ATM mutation, colorectal cancer, targeted NGS, case report

## Abstract

Poly-ADP ribose polymerase (PARP) inhibitors are constantly increasing in their indications for use as anti-cancer treatment in various neoplasms, the majority of which are linked with BRCA deficiency. Preclinical data support the investigation of PARP inhibitors in other neoplasms exhibiting “BRCAness” or homologous recombination deficiency (HRD) as monotherapy as well as in combination with chemotherapy. With the current report we present the case of a heavily pretreated 55-year-old male patient diagnosed with stage IV ATM-deficient CRC, who was effectively treated with an off-label olaparib-irinotecan combination after exhaustion of all available treatment choices; furthermore, we discuss the existing data providing evidence for the use of PARP inhibitors in ATM-deficient CRC and encourage the implementation of next-generation sequencing (NGS) in patients with no other available treatment options.

## Introduction

Colorectal carcinoma (CRC) is currently the 3^rd^ most common diagnosed malignancy in the United States of America. Prognosis is excellent for early-stage disease, but only 3% of patients with stage IV disease at diagnosis achieve a 5-year survival. Various drug categories – including chemotherapy, antiangiogenics and immune checkpoint inhibitors – based mainly on targeted driver oncogene mutation sequencing and immunohistochemistry - are being employed in the treatment of extensive disease (1).

Recent advances in the field of molecular oncology with the application of NGS have allowed a personalized approach in medical oncology and could contribute to exploring further treatment choices when current approved options are exhausted, especially for patients who maintain a good performance status (2).

With this report, we introduce the case of a heavily pretreated 55-year-old male patient, who was diagnosed with ATM-deficient CRC and whose treatment with an off-label olaparib – irinotecan combination led to radiographic disease stabilization, decrease in tumor markers and improvement of performance status.

## Case Description

A 51-year-old male patient was diagnosed with a stage IV colorectal (CRC) adenocarcinoma with multiple liver metastases in August 2017.

He gradually developed constipation during the past few months. Imaging revealed a 3cm mass in the area of the splenic flexure causing coprostasis, as well as multiple liver metastases with a maximal diameter of 17.3mm. Of note, a 6mm nodule was also found in the left upper pulmonary lobe. At that time, the patient received no medication, exercised regularly and was trying to quit smoking (18 pack-years) He reported social alcohol consumption. Familial history was quite suspicious for hereditary cancer syndromes; his mother was diagnosed with breast cancer at the age of 60 and with lung cancer at the age of 32, his maternal aunt and her daughter with breast cancer at young age, and his paternal grandmother with colorectal cancer at the age of 85.

An attempt to perform colonoscopy was unsuccessful due to episodes of vomiting, which raised the surgeons’ suspicion of intestinal obstruction, and therefore he underwent a right colectomy in August 2017. Histology revealed a low- to intermediate- grade T4aN2a colorectal adenocarcinoma– microsatellite stable (MSS), K-RAS mutated, B-RAF wild-type, and N-RAS wild-type.

From September 2017 to April 2021, the patient received various chemotherapeutic regimens, including capecitabine-oxaliplatin-bevacizumab (bevacizumab-XELOX), FOLFIRI-aflibercept, aflibercept-trifluridine/tipiracil (Lonsurf^®^), and FOLFIRINOX. Meanwhile, he underwent radiofrequency ablation (RFA) to the metastatic hepatic lesions in May 2018, but soon after that he developed bilateral lung metastases.

Due to the lack of approved treatment choices, we performed somatic NGS; NM_000051.3:c.8925_8928dup: p.(Glu2977Argfs*2) and NM_000051:c.3880dup: p.(Ile1294Asnfs*8) genetic alterations were found in Ataxia-telangiectasia mutated (ATM) gene, while K-RAS NM_033360.4:c.38G>A:p.(Gly13Asp) alteration was also present. Both of these ATM mutations are considered unique based on the COSMIC database and thus their effect on functional relevance is unknown (3), but the mutation at amino acid 2977 results in a premature termination codon and may thus be considered deleterious (4). A different mutation at amino acid 1294 has been observed and is considered pathogenic based on FATHMM prediction score (5). Interestingly, a parallel constitutional exome analysis revealed the presence of ATM NM_000051.3:c.8925_8928dup: p.(Glu2977Argfs*2) mutation in heterozygosity, which is inherited as autosomal dominant. This variant was considered pathogenic based on the criteria of the American College of Medical Genetics and Genomics and the Association for Molecular Pathology (6), so we suggested that our patient’s children as well as his sister should consult a genetician due to the high risk for breast, ovarian and other malignancies (7).

Based on and the findings from NGS, we searched the literature for noteworthy targetable choices and concluded that there is rationale for targeting our patient’s ATM mutation with a combination of PARP inhibition and chemotherapy. The only clinical study performed in CRC patients is a phase I Canadian study, which employed olaparib and irinotecan in unselected pretreated CRC patients proving the safety of this combination and leading to SD in the majority of patients, so we applied for this combination in April and received approval from the National Drug Organization in May 2021 (8). The current data regarding the position of PARP inhibitors in treatment of CRC is extensively described in the “Discussion” section below.

**Figure 1:**
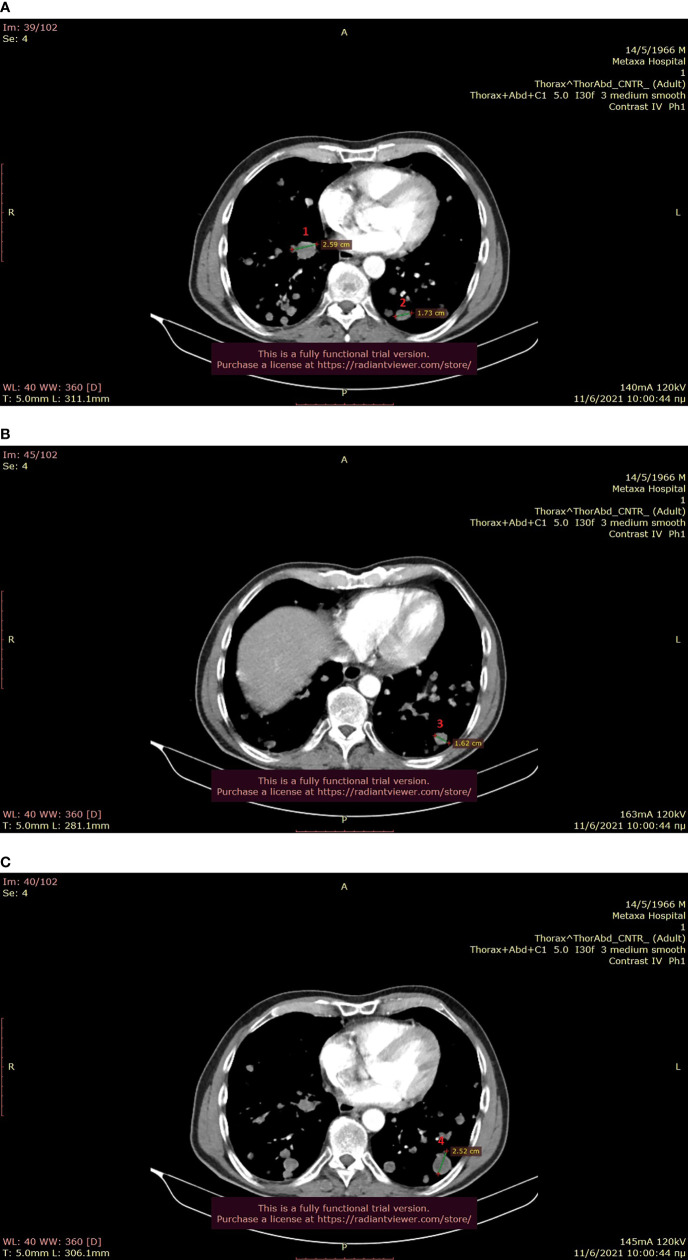
**(A–C)**: Baseline thoracic CT showing multiple metastases bilaterally (June 2021).

**Figure 2:**
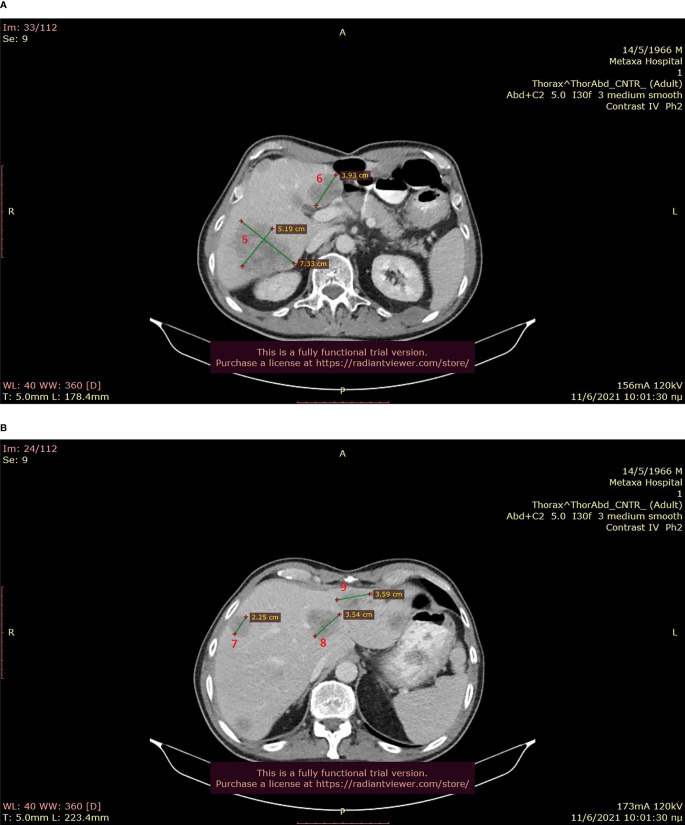
**(A, B)**: Baseline abdominal CT showing multiple hepatic metastases (June 2021).

In anticipation of approval, our patient received pembrolizumab – regorafenib during April and May 2021, but he tolerated regorafenib poorly and showed early signs of immune-mediated hepatotoxicity, so this combination was abandoned early. He was vaccinated against COVID-19 in May 2021, and we chose to initiate the off-label approved olaparib – irinotecan combination in June 2021. Patient’s performance status was 2 at that time, he weighed 64 kg and suffered from paroxysmal dry cough, pain in the upper right abdominal area due to his hepatic disease that was well-controlled with opioids, and episodes of incomplete intestinal obstruction associated with a large peritoneal metastasis laying in contact with the bowel as well as with signs of localized PD in the large intestine (**Figures 1A–C**, **2A, B**). His medication included lansoprazole 30mg od, dexamethasone sir 4mg od, fentanyl 12μg/hr transdermally, fentanyl 100 UG as per needed orally, ipratropium and budesonide inhalations, metoprolol 50mg bid, levothyroxine 12mcg od, paracetamol/codeine/caffeine per os as per needed for pain control, metoclopramide sir as per needed against nausea and paraffin oil for constipation.

**Figure 3:**
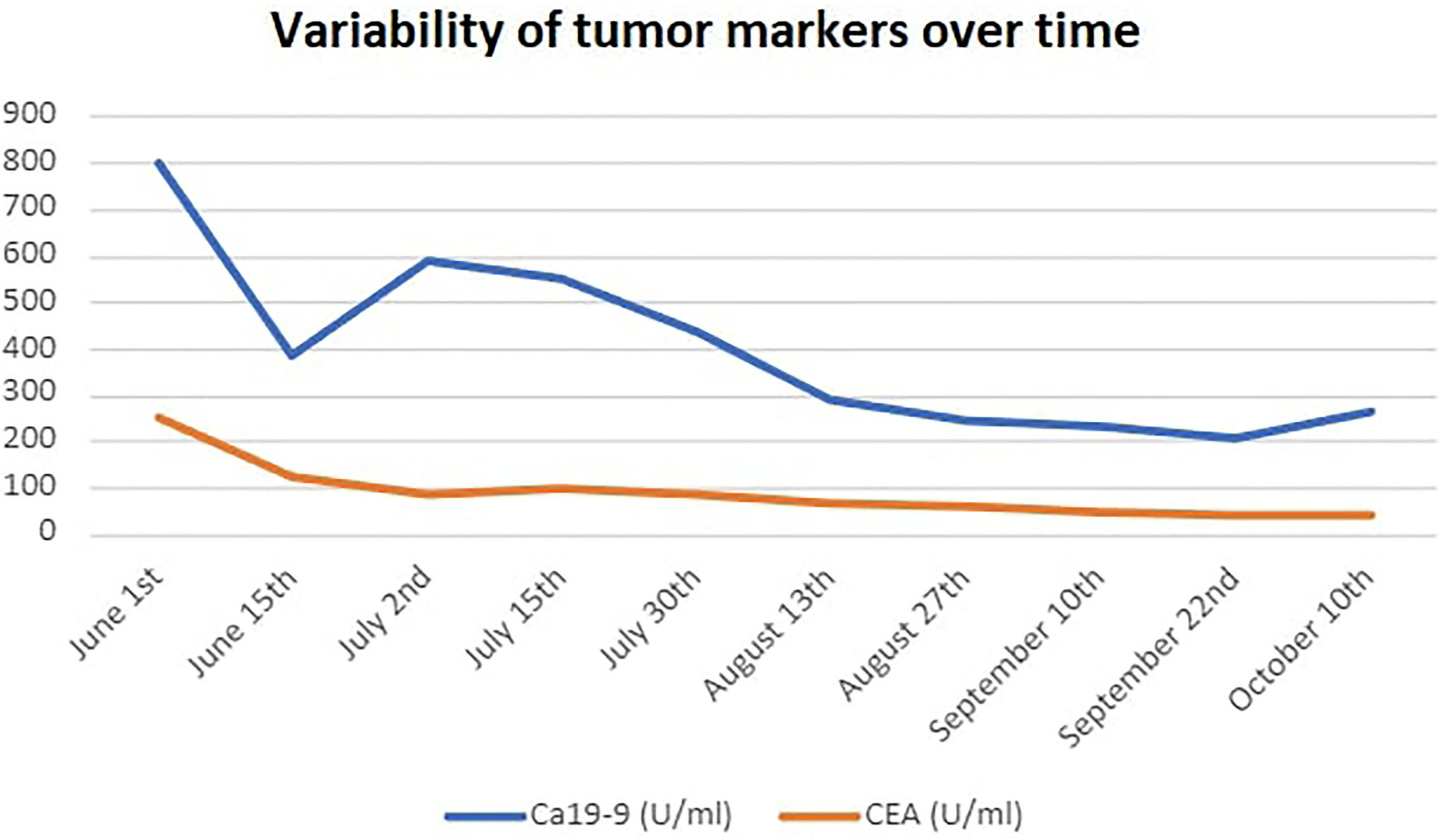
Variability of tumor markers over time.

**Figure 4:**
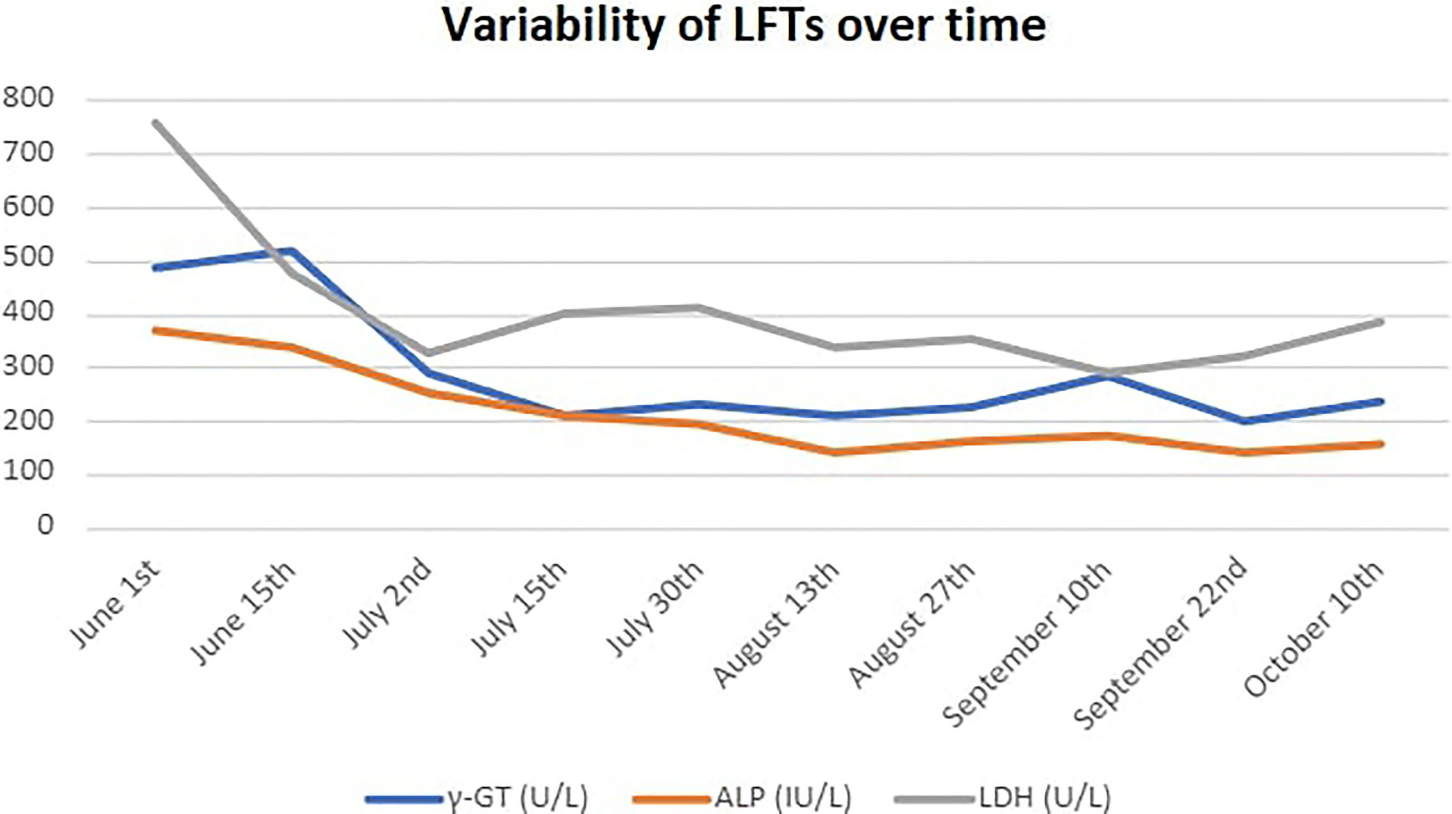
Variability of liver function tests (LFTs) over time.

Before starting combination treatment, we discussed with the patient about the rationale for our choice, emphasized the fact that this is an off-label treatment combination and obtained informed consent. Treatment protocol included irinotecan as intravenous infusion at 125mg/m^2^ (on day 1) and olaparib 50mg capsules given bid (days 1-5) every 2 weeks (8). G-CSF support was needed at days 7-9 of each course. His tumor markers started to decrease rapidly from the 1^st^ course (Ca19-9: 390 U/ml and CEA: 127 U/ml on the 15^th^ of June from baseline 800 U/ml and 127 U/ml, respectively on the 1^st^ of June) (**Figure 3**). He was able to stop steroids in the 3^rd^ course, while constipation had gradually resolved. We added mirtazapine 15mg od in order to assist his appetite, and transdermal granisetron to manage treatment-related nausea. His liver function tests (LFTs), that were somewhat affected during pembrolizumab – regorafenib treatment also started to decrease (**Figure 4**), and he managed to gain 4kgs in August 2021. His performance status was also improved to 0, his dry cough started to subside, and he had no complaints of pain, so we decided to stop fentanyl.

**Figure 5:**
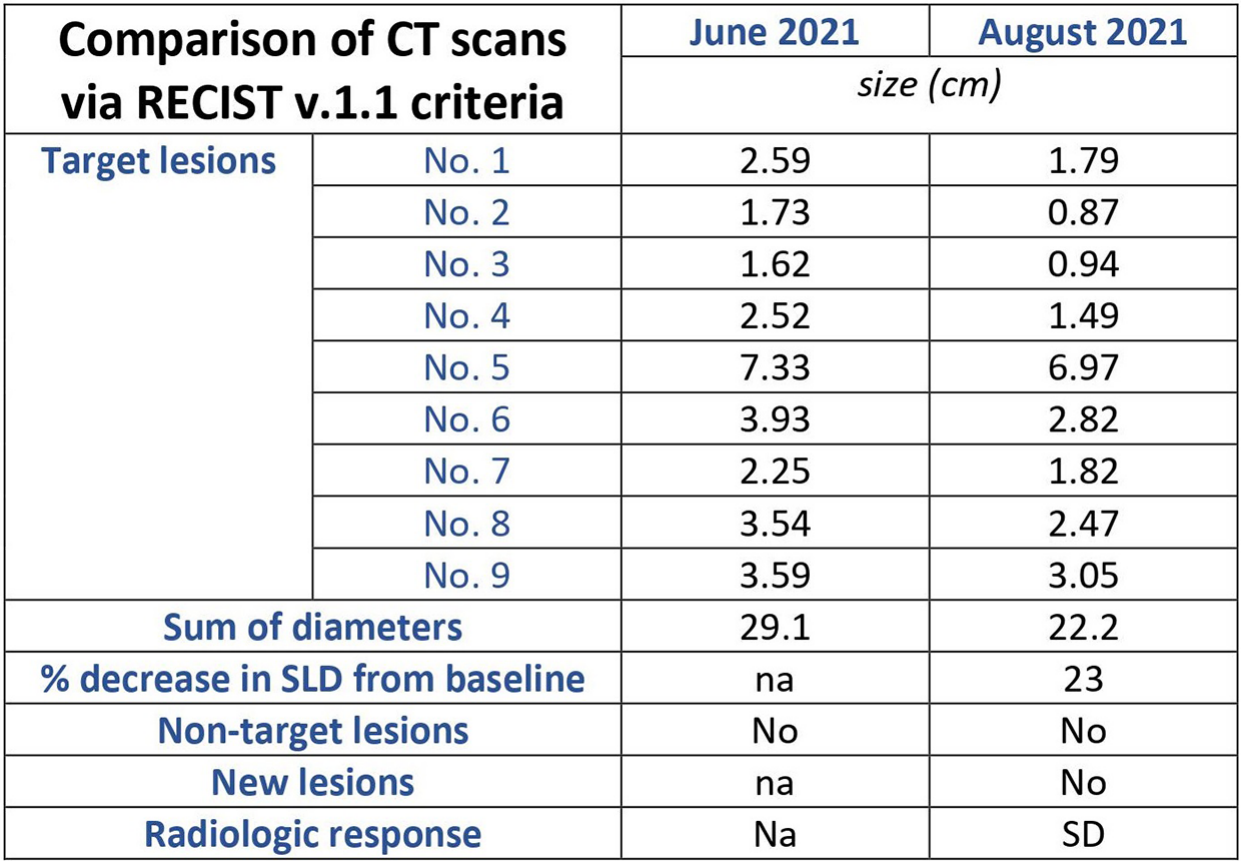
Comparison of CT scans via RECIST v.1.1 criteria. na, not applicable.

**Figure 6:**
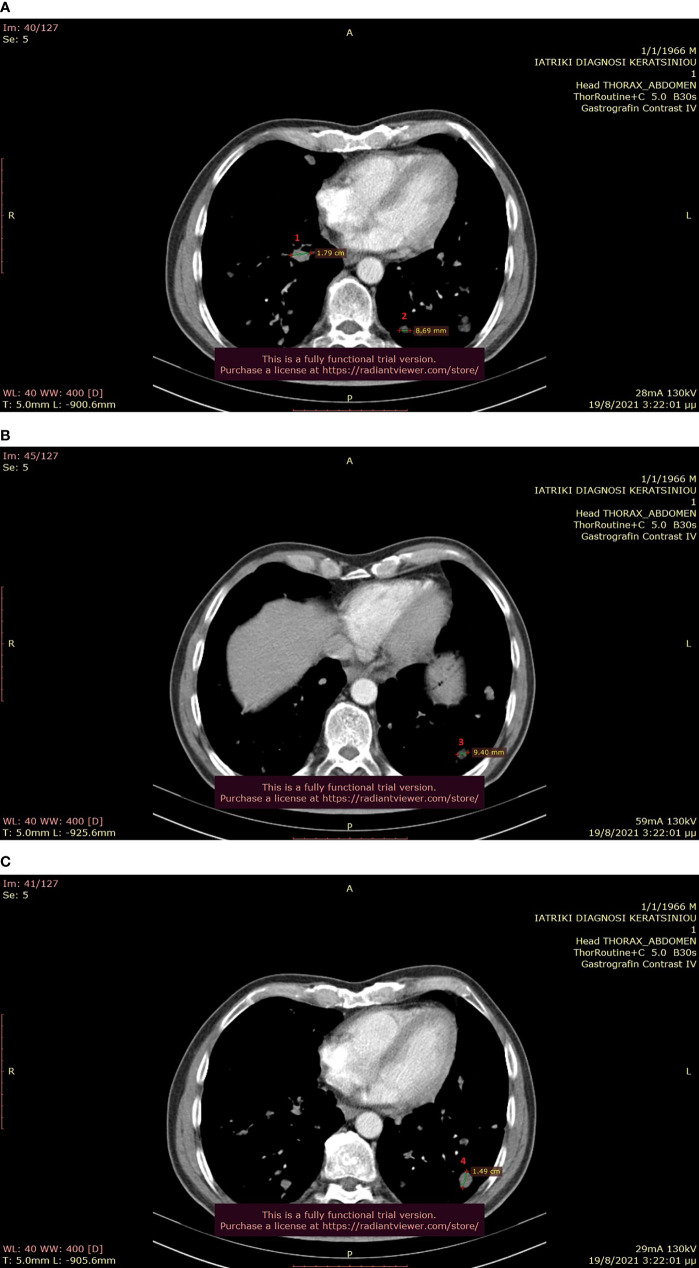
**(A–C)**: Thoracic CT after the completion of 6 treatment courses, showing SD in the lung (August 2021).

**Figure 7:**
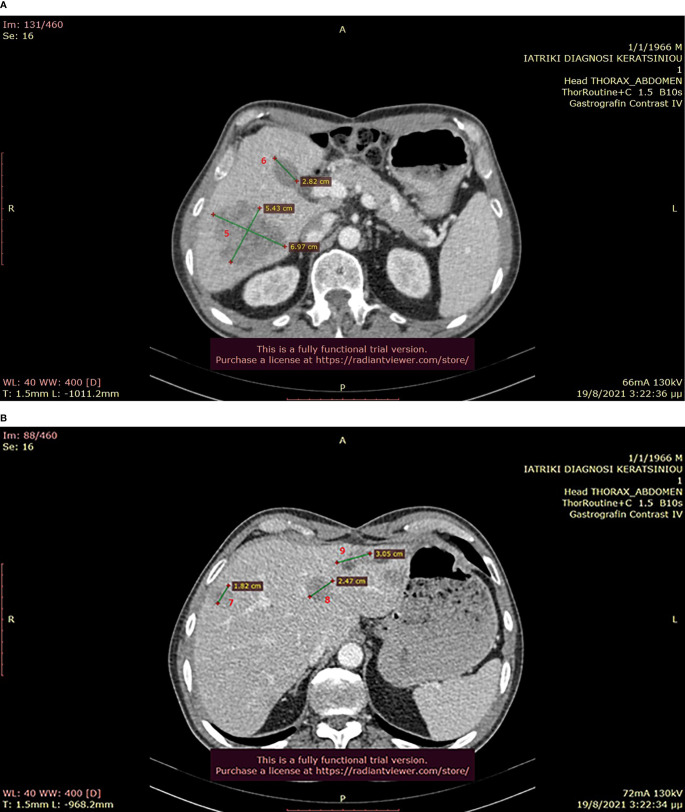
**(A, B)**: Abdominal CT after the completion of 6 treatment courses, showing SD in the liver (August 2021).

Imaging reevaluation after the 6^th^ course revealed SD according to Response Criteria for Solid Tumors (RECIST) version 1.1 (**Figure 5**) (9) in the pulmonary (**Figures 6A–C**), as well as in the abdominal disease (**Figures 7A, B**), while tumor markers continued to decrease. Our patient tolerated treatment well and experienced a clinical response that lasted 4 months, which allowed him to live a social life again. Unfortunately, his disease progressed again in October 2021, but his performance status is still unaffected.

## Discussion

ATM is a major regulator for DNA damage repair (DDR) mechanisms responding to double-strand breaks, while the loss of its activity is associated with increased risk for various malignancies, including CRC (10). The serine/threonine kinase ATM is operating in the early stages of homologous recombination mechanism, causing cell-cycle arrest *via* TP53 and to repair of DNA damage *via* activation of BRCA 1/2 (11). Mutations in ATM are present in 9% of CRC, the majority of which are missense (12). Germline heterozygous ATM alterations are found in 1% of general population and are linked with an increased incidence of cancer (13).

Regarding our patient, preclinical data support the correlation of ATM deficiency with better prognosis in CRC (14). Somatic mutations may induce resistance to chemotherapy (15), while germ-line mutations seem to correlate with increased chemotherapeutic toxicity (15). During the last years, a wide effort to capitalize on DDR mutations towards a shift to personalized treatment has been initiated; platinum drugs, PARP inhibitors, ATR inhibitors and checkpoint inhibitors are in the center of attention (15, 16).

PARP inhibitors are known to be effective in BRCA-deficient tumors (17) and are FDA-indicated in ovarian, fallopian tube, primary peritoneal, breast cancer (18), as well as pancreatic (19) and prostate cancer (20). There is also preclinical evidence of PARP inhibitors’ effectiveness in tumors carrying somatic mutations in other DNA repair genes, such as ATM, ATR, CHECK2, PALB2 and RAD51 (21). Olaparib seems to act as cytostatic and not cytotoxic in ATM-deficient tumors (22) and also has potential in ATM-deficient CRC (23).

Indeed, a phase II study of olaparib monotherapy in chemo-refractory CRC patients failed to provide clinical efficiency, both in MSI-high and in MSI-low tumors and was prematurely terminated (24). A French team reported on 2 heavily pretreated patients with homologous repair damage (HRD) deficient CRC that were treated with olaparib based on the findings of NGS analysis; one patient with a Check2 mutation responded well to treatment, but the other one with a RAD51C mutation had PD that was attributed to a frameshift truncating mutation in the TP53BP1 gene. The same report provides three possible mechanisms of resistance to PARP inhibitors; restoration of BRCA1 function by additional alterations, increased expression of Mdr1 gene and loss of TP53BP1 (25). Unfortunately, we are still unable to identify which ATM-deficient tumors may benefit from PARP inhibition, so direct testing on organoids or patient-derived xenografts might be useful (26).

To date, the only clinical trial evaluating a possible synergy between a PARP inhibitor and chemotherapy in CRC is a phase I Canadian study from 2016. More specifically, this study aimed to investigate the use of olaparib – irinotecan combination in unselected pretreated CRC patients. 9 out of 25 enrolled patients achieved SD, while it was proven that intermittent olaparib is better tolerated in comparison to continuous administration due to pharmacodynamic interaction with chemotherapy. The study concluded that the recommended phase II dosages were olaparib 50mg per os twice daily (days 1-5) and irinotecan 125mg/m^2^ (day 1) every two weeks (8).

Furthermore, there is encouraging data for synergistic activity of olaparib with oxaliplatin in CRC (27–30). A recent translational study pointed out that there is cross-sensitivity between olaparib and oxaliplatin in B-RAF- and K-RAS-mutated CRC cells, whilst no cross-sensitivity between olaparib and 5-fluoruracil was found. The same study suggested that sequential olaparib after first-line oxaliplatin-based treatment may prolong progression free survival; thus, response to oxaliplatin may define ‘‘clinical BRCAness’’ in CRC (31, 32). 5-fluoruracil – olaparib combination has also offered notable results in MMR-deficient CRC in the preclinical setting (32).

## Conclusion

Given the above mentioned advances in the preclinical setting, we assume that a consequent phase II study for patients with ATM-deficient CRC should be conducted, as the olaparib – irinotecan combination seems to be well tolerated even in heavily pretreated patients, similar to our case. The oxaliplatin – olaparib combination may also be worth to study.

Preclinical data also suggest the investigation of PARP inhibitors as maintenance treatment after response to oxaliplatin in CRC. Indeed, the LYNL-003 trial is a phase III study evaluating a possible superiority of olaparib with or without bevacizumab versus bevacizumab – 5-fluoruracil as maintenance treatment after first-line FOLFOX – bevacizumab (NCT04456699, recruiting). Furthermore, the trend of combining PARP inhibitors with immunotherapy is emerging, as PARP inhibitors have been shown to introduce mutations by inducing double-strand breaks (DSB), which will ultimately promote neo-antigen generation, to increase tumor mutational burden and to enhance the expression of PD-L1 (33).

In any event, it is valuable to perform NGS in pretreated patients that lack effective treatment choices, in order to explore a possible personalized approach that could ultimately increase overall survival and preserve a good quality of life.

## Data Availability Statement

The original contributions presented in the study are included in the article/supplementary material. Further inquiries can be directed to the corresponding author.

## Ethics Statement

Written informed consent was obtained from the individual(s) for the publication of any potentially identifiable images or data included in this article.

## Author Contributions

GP treated the patient, analyzed the data, and wrote the manuscript. EF acquired, analyzed, and interpreted the data. NS acquired, analyzed, and interpreted the data. EC acquired, analyzed, and interpreted the data. ST acquired, analyzed, and interpreted the data. EL acquired, analyzed, and interpreted the data. CK treated the patient, wrote the manuscript, and supervised this manuscript. All authors contributed to the article and approved the submitted version.

## Conflict of Interest

The authors declare that the research was conducted in the absence of any commercial or financial relationships that could be construed as a potential conflict of interest.

## Publisher’s Note

All claims expressed in this article are solely those of the authors and do not necessarily represent those of their affiliated organizations, or those of the publisher, the editors and the reviewers. Any product that may be evaluated in this article, or claim that may be made by its manufacturer, is not guaranteed or endorsed by the publisher.
